# Evaluation of cardiac function at different time points after myocardial infarction of rats

**DOI:** 10.1186/1479-5876-10-S2-A58

**Published:** 2012-10-17

**Authors:** Huiyuan Hu, Yan Zhao, Shuang Liu, Wang Yan, Aosiman Yushupujiang, Sitong Jin, Xuefei Sun, Weidong Ren, Liying Hao

**Affiliations:** 1 Dept. of Pharmaceutical Toxicology, China Medical Univ., Shenyang 110001, China; 2 Dept. of Cardiovascular funciton, China Medical Univ., Shenyang 110001, China

## Backgrounds

Myocardial infarction (MI) model of rats is commonly used in cardiovascular research. However, the noninvasive functional evaluation parameters in this model are lack. Echocardiography is an important tool for assessment of cardiac function in clinic. This study is to explore whether changes of the cardiac function after MI of rats could be detected by echocardiography accurately.

## Methods

MI were induced in rats by ligating the left anterior descending coronary artery. Cardiac function and pathological changes were measured by Dimension echocardiography and transmission electron microscope (TEM) at 24h, 1w, 2w and 4w after MI, respectively.

## Results

Compared with sham group, cardiac function did not change in 24h group after ligation, but the interventricular septum thickness at end-systole and interventricular septum thickness at end-diastole in groups of 1, 2, 4 weeks after operation were significantly decreased (*P*<0.05); the left ventricular internal dimension at end-systole in groups of 1, 4 weeks after MI and left ventricular internal dimension at end-diastole in 4w group were dramastically increased (*P*<0.05); the ejection fraction and left ventricular fraction shortening in groups of 1, 2, 4 weeks after MI declined significantly (*P*<0.05); in addition, the mitral valve E peak in 2w group was greatly reduced (*P*<0.05). These data suggested that both left ventricular systolic and diastolic function were affected 1 week after MI, with changes of cardiac sturcture. Moreover, TEM illustration showed that nuclear pyknosis, myofilament disruption, mitochondrial swelling were present in all MI groups and severe reconstruction and fibrosis were observed especially in 4w-MI group.

## Conclusion

Although cardiac structure was damaged 1 day after MI, its funciton was unchaged due to compansatory effect of normal tissues. With time goes on, the infarcted cardiac tissue was severely deformed and cardiac function was decreased accordingly. Ecochardiography could detect the changes of cardiac funciton accurately after MI, which will provide important experimental evidences for the clinical diagnosis, treatments and prognostic assements of MI patients.

